# Massive increase in monocyte HLA-DR expression can be used to discriminate between septic shock and hemophagocytic lymphohistiocytosis-induced shock

**DOI:** 10.1186/s13054-018-2146-2

**Published:** 2018-09-11

**Authors:** Solenn Remy, Morgane Gossez, Alexandre Belot, Jack Hayman, Aurelie Portefaix, Fabienne Venet, Etienne Javouhey, Guillaume Monneret

**Affiliations:** 1 Hospices Civils de Lyon, Paediatric Intensive Care Unit, Mother and Children University Hospital, 59 Boulevard Pinel, 69500 Bron, France; 2 Hospices Civils de Lyon, Immunology Laboratory, E. Herriot Hospital, 69003 Lyon, France; 3 EA 7426, Pathophysiology of injury-induced immunosuppression (University Claude Bernard Lyon 1, BioMérieux, Hospices Civils de Lyon), E. Herriot Hospital, 69003 Lyon, France; 4 Hospices Civils de Lyon, Paediatric Nephrology, Rheumatology, Dermatology Unit, National Referee Centre for pediatric-onset Rheumatism and autoimmune diseases (RAISE), Mother and Children University Hospital, 59 Boulevard Pinel, 69500 Bron, France; 5 Université de Lyon, INSERM U1111, CIRI, Lyon, France; 6 EPICIME-CIC 1407 de Lyon, Inserm, Service de Pharmacologie Clinique, CHU-Lyon, Bron, France; 7 Cellular Immunology Laboratory, Hôpital E. Herriot – Hospices Civils de Lyon, France Pavillon E – 5 place d’Arsonval, 69437 Lyon, Cedex 03 France

**Keywords:** Immune response, Septic shock, Biomarker, Hemophagocytic lymphohistiocytosis

Clinical presentations of hemophagocytic lymphohistiocytosis (HLH) and septic shock share many similarities, including multiple organ dysfunction and overall clinical and biological symptoms. However, these life-threatening conditions require specific and opposing treatments. Currently, no single biomarker is available to differentiate septic shock from HLH at patient admission [1, 2]. HLH is classified as a primary (genetically inherited) or a secondary, i.e., induced by various inflammatory conditions (viral infections, autoimmune processes, lymphoid malignancies, or drug allergies), immune disorder. We report here the case of a young woman with febrile shock which proved to be a HLH caused by drug-induced hypersensitivity syndrome (DIHS). As septic shock was initially suspected, the patient benefited from broad immunological screening during the first week of evolution [3]. Strikingly, this revealed massively increased expression of monocyte human leukocyte antigen-DR (mHLA-DR) at 137,021 ABC (antibody bound per cell), even though expected values in septic shock are usually drastically decreased [3]. In addition, a positive response to increasing doses of corticosteroids was observed over time (Fig. 1). More precisely, while the patient’s mHLA-DR expression was measured at 137,021 ABC at admission, it decreased to 38,961 ABC after the introduction of corticosteroids (day 3). Following the reactivation of inflammatory processes (day 5), mHLA-DR rose again (66,829 ABC). Finally, mHLA-DR returned to a normal range after increasing corticosteroid doses (20,499 ABC, day 8). All clinical features are provided in Additional file 1.

**Fig. 1:**
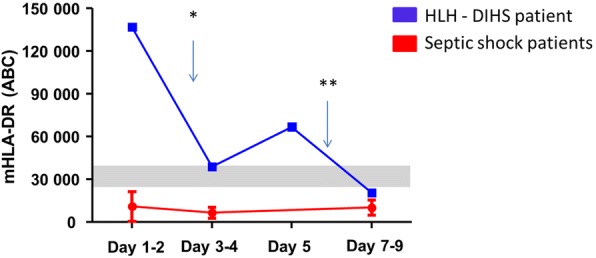
Time course of mHLA-DR in a HLH patient. *Blue squares* depict mHLA-DR values in the HLH patient. *Red circles* represent pediatric septic shock values [3]. *Gray range* represents interquartile range values obtained previously in healthy children [3]. *Corticosteroids introduced, receiving 2 mg/kg/day; **corticosteroid adjustment to 4 mg/kg/day

In the present patient, the extremely increased inaugural mHLA-DR value (i.e., 137,021 ABC) helped to unequivocally exclude a diagnosis of septic shock. Indeed, in our experience (more than 600 septic shock patients monitored over several years), the vast majority of mHLA-DR values measured within the first 3 days after septic shock are reported to be < 30,000 ABC and mostly found below 10,000 ABC (normal values ranged from 15,000 to 40,000 ABC). This agrees with pathophysiology since HLH is secondary to overproduction of interferon-γ (IFN-γ), a cytokine known to be a strong inducer of mHLA-DR expression, whereas sepsis induces downregulation of mHLA-DR expression.

In conclusion, mHLA-DR may discriminate septic shock from HLH at admission despite both situations with multiple organ dysfunction sharing very common clinical and biological features (e.g., sCD25, elevated ferritin levels) [4, 5]. This result obviously needs further assessment in various types of HLH. Upon confirmation, as these two deadly conditions (i.e., septic shock and HLH) would require opposing treatments, mHLA-DR may be of crucial help for clinicians regarding patients’ care and management.

## Additional file

Additional file 1: Additional online information. (DOCX 27 kb)

## Abbreviations

ABC: Antibody bound per cell
DIHS: Drug-induced hypersensitivity syndrome
HLH: Hemophagocytic lymphohistiocytosis
IFN-γ: Interferon-γ
mHLA-DR: Monocyte human leukocyte antigen-DR
