# Supplementary material for: Massive increase in monocyte HLA-DR expression can be used to discriminate between septic shock and hemophagocytic lymphohistiocytosis-induced shock
Source: Crit Care. 2018 Sep 11;22:213. doi: 10.1186/s13054-018-2146-2 (PMC6131803; doi:10.1186/s13054-018-2146-2)
Supplement: Supplementary file 1 — Additional online information. (DOCX 27 kb) [file 13054_2018_2146_MOESM1_ESM.docx]

**Additional file 1**

**Massive increase in monocyte HLA-DR expression can be used to discriminate between septic shock and hemophagocytic lymphohistiocytosis-induced shock**

Solenn REMY ^1^, Morgane GOSSEZ ^2^, Alexandre BELOT ^3 4^, Jack Hayman ^2^, Aurelie PORTEFAIX ^5^, Fabienne VENET ^2 6^, Etienne JAVOUHEY ^1^, Guillaume MONNERET ^2 6^

**Affiliations:**

^1^ Hospices Civils de Lyon, Paediatric Intensive Care Unit, Mother and Children University Hospital, 59 Boulevard Pinel, 69500 Bron, France

^2^ Hospices Civils de Lyon, Immunology Laboratory, E. Herriot Hospital, Lyon, 69003, France

^3^ Hospices Civils de Lyon, Paediatric Nephrology, Rheumatology, Dermatology Unit, National Referee Centre for pediatric-onset Rheumatism and autoimmune diseases (RAISE), Mother and Children University Hospital, 59 Boulevard Pinel, 69500 Bron, France

^4^ Université de Lyon, INSERM U1111, CIRI, Lyon France

^5^ EPICIME-CIC 1407 de Lyon, Inserm, Service de Pharmacologie Clinique, CHU-Lyon, Bron, France

^6^ EA 7426, Pathophysiology of injury-induced immunosuppression, University Claude Bernard Lyon 1, BioMérieux Hospices Civils de Lyon, E. Herriot Hospital, Lyon, 69003, France

**Corresponding author:**

Guillaume MONNERET Cellular Immunology Laboratory, Hôpital E. Herriot – Hospices Civils de Lyon, France Pavillon E – 5 place d’Arsonval 69437 LYON Cedex 03 – France guillaume.monneret@chu-lyon.fr Tel: +33 4 72 11 97 58

**Patient information and clinical findings**

A 16-year-old girl was admitted to our Pediatric Intensive Care Unit (PICU), Lyon University Hospital, France, with shock and multi-system organ failure. She had a medical history of focal cryptogenic epilepsy started five years earlier, treated by valproic acid for years, recently replaced by lamotrigine. Eighteen days after lamotrigine initiation, she went to a local emergency unit for fever, rash and hypotension. She was in menstrual condition, with the same absorbency tampon used for 12 hours. She was hospitalized in adult ICU with Toxic Shock Syndrome as first diagnosis, treated by amoxicillin clavulanic acid and clindamycin, and required hemodynamic support by norepinephrine for 2 days. Due to rash and hypotension, a Drug-Induced Hypersensitivity Syndrome (DIHS) to lamotrigine was also hypothesized. Therefore, lamotrigine was replaced with levetiracetam. No corticosteroid was introduced. Favorable outcome allowed hospital discharge after 3 days.

Two days after returning home, she presented again with arterial hypotension, skin rash and fever. She was transferred from emergency unit to our PICU for febrile shock without obvious etiological diagnosis. Admission in PICU is considered as day 1 of care (figure 1). Clinically, she presented with vasoplegic shock associated with altered consciousness, fever at 40°C and a measles-like rash on chest and face. Biologically, she showed metabolic acidosis, renal failure, systemic inflammation, hepatic and hematologic dysfunctions (pH = 7.36, lactate = 4.4 mmol/L; urea = 7.2 mmol/L, creatinine = 111 µmol/L; C-reactive protein = 30 mg/L, procalcitonin = 33.6 µg/L; Aspartate Aminotransferase (AST) = 1,281 UI/L, Alanine Aminotransferase (ALT) = 666 UI/L; INR = 2.2 , prothrombin time (PT) = 25 %, fibrinogen = 1.83 g/L). Complete Blood Count was slightly disturbed, without hypereosinophilia but with activated lymphocytes (hemoglobin = 115 g/L, platelets = 140 G/L, White Blood Cells = 12.08 G/L, neutrophils = 6.64 G/L, lymphocytes = 4.83 G/L, eosinophils = 0.48 G/L). She underwent intubation and mechanical ventilation with continuous sedation and received norepinephrine up to 1 μg/kg/h.

Broad-spectrum antibiotics were given for two supplemental days (ceftriaxone, amikacin and spiramycin). Hydrocortisone (1mg/kg/ 6 hours) and intravenous immunoglobulins (2 g/kg) were added for vasopressor refractory shock.

At day 2, hemophagocytic lymphohistiocytosis (HLH) was established: there were 97 % probability according to HScore (224 points)^1^ and five criteria fulfilled according to HLH-2004 diagnostic guidelines^2^: fever, splenomegaly, increased ferritin up to 3,918 µg/L, hypofibrinogenemia (0.47 g/L), two cytopenias (platelets = 25 G/L and hemoglobin = 83 g/L on day 3). NK cell activity revealed normal degranulation (CD107 expression upon stimulation) and membrane perforin expression (controlled on 2 measures by flow cytometry). Moreover, the medullogram excluded lymphoma and showed eosinophilia with activated lymphocytes without hemophagocytosis. DIHS etiology for secondary HLH was retained, according to the following criteria: (i) lamotrigin imputability with compatible chronology, lack of microbiology identification. (ii) Skin biopsy confirmed a drug-induced dermatitis with eosinophilic infiltrate. (iii) Numerous lymphadenopathies with splenomegaly were identified by computed-tomography. (iiii) Biological abnormalities: delayed eosinophilia at 2.0 G/L appeared at day 3 associated with activated lymphocytosis in blood. Total RegiSCAR was on 9^3^. No viral reactivation (including HHV-6) was found on several samples. Consequently, corticosteroids were introduced (2 mg/kg/day), leading to a rapid improvement (extubation, norepinephrine stop, rash disappearance, apyrexia). Unfortunately, at day 5, she presented with a further worsening characterized by hypotension, fever, skin rash reappearance, hepatic cytolysis recurrence and a pericarditis. Corticosteroids were increased up to 4 mg/kg/day, allowing overall improvement: and a discharge from PICU and pediatric unit after 10 and 17 days, respectively.

Reactive hemophagocytic syndrome is an unregulated hyper-inflammatory condition caused by various triggers: infections (Epstein-Barr virus, leishmanioses…), hematological malignancies and autoimmune diseases (juvenile idiopathic arthritis, Still’s disease…). DIHS is also a multi-organ systemic reaction which shares overlapping symptoms with HLH. Even though pathophysiological mechanisms are lacking, DIHS has been described as a possible HLH trigger, probably through lymphocytes and macrophages activation^4-6^.

**Immune response**

Because of initial presentation, with suspected septic shock or toxic shock syndrome, the patient was included in a clinical research protocol entitled PedIRIS study (Pediatric Immune Response to Infectious Shock, NCT 02848144). This was a monocentric observational study, including children between 1 month and 18 years old, admitted to PICU for an infectious shock. Blood was sampled at day 1-2, 3-5 and 7-9 after shock onset. Immune monitoring included monocyte Human Leukocyte Antigen-DR (mHLA-DR), lymphocytes subsets’ counts and concentrations of following cytokines: interleukine-6, interleukine-8, interleukine-10, interleukine-1 receptor antagonist, tumor necrosis factor-α and interferon-γ.

As presented in the letter, the patient revealed an unexpected massive expression of mHLA-DR. Another interesting point concerned regulatory T cells. Their percentages among total CD4⁺ lymphocyte population were collapsed at each time point: 1.1 % (day 1), 2.1 % (day 3), 0.8 % (day 5) and 1.7 % (day 8), while median observed proportions in septic patients and healthy children were respectively 7.24 % and 6.8 % ^7^. We did not observe differences with septic patients regarding other lymphocytes subsets and cytokines levels.

**References**

1. Fardet L, Galicier L, Lambotte O, et al. Development and validation of the HScore, a score for the diagnosis of reactive hemophagocytic syndrome. Arthritis and Rheumatology 2014; 66:2613-2620.
2. Henter Jl, Horne A, Arico M, et al. HLH-2004: diagnostic and therapeutic guidelines for hemophagocytic lymphohistiocytosis. Pediatr Blood Cancer 2007; 48:124-31.
3. Kardaun SH, Sekula P, Valeyrie-Allanore L, et al. Drug reaction with eosinophilia and systemic symptoms (DRESS): an original multisystem adverse drug reaction. Results from the prospective RegiSCAR study. Br J Dermatol 2013; 169(5):1071-80.
4. Ben M’Rad M, Leclerc-Mercier S, Blanche P, et al. Drug-induced hypersensitivity syndrome: clinical and biologic disease patterns in 24 patients. Medicine (Baltimore) 2009; 88(3):131-40.
5. Penel-Page M, Ben Said B, Phan A, et al. Correctly adDRESS the cause of hemophagocytic lymphohistiocytosis. Arch Pediatr. 2017 Mar;24(3):254-259.
6. Picard M, Fernandez MI, Des Roches A, et al. Ceftazidime-induced drug reaction with eosinophilia and systemic symptoms (DRESS) complicated by hemophagocytic lymphohistiocytosis. J Allergy Clin Immunol Pract. 2013 Jul-Aug;1(4):409-12.
7. Remy S, K.-D. K. (2018). Occurrence of marked sepsis-induced immunosuppression in pediatric septic shock: a pilot study. Ann Intensive Care, 8(1):36.
